# Molecular Characterisation of a Rare Reassortant Porcine-Like G5P[6] Rotavirus Strain Detected in an Unvaccinated Child in Kasama, Zambia

**DOI:** 10.3390/pathogens9080663

**Published:** 2020-08-17

**Authors:** Wairimu M. Maringa, Peter N. Mwangi, Julia Simwaka, Evans M. Mpabalwani, Jason M. Mwenda, Ina Peenze, Mathew D. Esona, M. Jeffrey Mphahlele, Mapaseka L. Seheri, Martin M. Nyaga

**Affiliations:** 1 Next Generation Sequencing Unit, Division of Virology, Faculty of Health Sciences, University of the Free State, Bloemfontein 9300, South Africa; makena96wairimu@gmail.com (W.M.M.); nthigapete@gmail.com (P.N.M.); 2 Virology Laboratory, Department of Pathology & Microbiology, University Teaching Hospital, Adult and Emergency Hospital, Lusaka 10101, Zambia; juliachibumbya@gmail.com; 3 Department of Paediatrics & Child Health, School of Medicine, University of Zambia, Ridgeway, Lusaka RW50000, Zambia; evans.mpabalwani@unza.zm; 4 World Health Organization, Regional Office for Africa, Brazzaville P.O. Box 06, Congo; mwendaj@who.int; 5 Diarrhoeal Pathogens Research Unit, Faculty of Health Sciences, Sefako Makgatho Health Sciences University, Medunsa, Pretoria 0204, South Africa; ina.peenze@smu.ac.za (I.P.); mathew.esona@gmail.com (M.D.E.); Jeffrey.Mphahlele@mrc.ac.za (M.J.M.); mapaseka.seheri@smu.ac.za (M.L.S.); 6 South African Medical Research Council, 1 Soutpansberg Road, Pretoria 0001, South Africa

**Keywords:** whole-genome, genotype constellation, interspecies transmission, reassortment, porcine, porcine-like human

## Abstract

A human-porcine reassortant strain, RVA/Human-wt/ZMB/UFS-NGS-MRC-DPRU4723/2014/G5P[6], was identified in a sample collected in 2014 from an unvaccinated 12 month old male hospitalised for gastroenteritis in Zambia. We sequenced and characterised the complete genome of this strain which presented the constellation: G5-P[6]-I1-R1-C1-M1-A8-N1-T1-E1-H1. The genotype A8 is often observed in porcine strains. Phylogenetic analyses showed that VP6, VP7, NSP2, NSP4, and NSP5 genes were closely related to cognate gene sequences of porcine strains (e.g., RVA/Pig-wt/CHN/DZ-2/2013/G5P[X] for VP7) from the NCBI database, while VP1, VP3, VP4, and NSP3 were closely related to porcine-like human strains (e.g., RVA/Human-wt/CHN/E931/2008/G4P[6] for VP1, and VP3). On the other hand, the origin of the VP2 was not clear from our analyses, as it was not only close to both porcine (e.g., RVA/Pig-tc/CHN/SWU-1C/2018/G9P[13]) and porcine-like human strains (e.g., RVA/Human-wt/LKA/R1207/2009/G4P[6]) but also to three human strains (e.g., RVA/Human-wt/USA/1476/1974/G1P[8]). The VP7 gene was located in lineage II that comprised only porcine strains, which suggests the occurrence of independent porcine-to-human reassortment events. The study strain may have collectively been derived through interspecies transmission, or through reassortment event(s) involving strains of porcine and porcine-like human origin. The results of this study underline the importance of whole-genome characterisation of rotavirus strains and provide insights into interspecies transmissions from porcine to humans.

## 1. Introduction

Group A rotaviruses (RVA), of the family *Reoviridae*, are the number one viral pathogens causing severe diarrhoea in children below five years of age [1]. In 2016, an estimated 128,000 deaths in children below five years were due to RVA infections, 90% of which occurred in developing countries [2,3]. Similarly, RVA are the primary cause of acute gastroenteritis in new-born piglets [4]. 

Rotaviruses have a distinctive morphology which comprises a nonenveloped, three-layered icosahedral protein shell. The rotavirus genome within the protein shell comprises 11 segments of double-stranded (dsRNA) that encode six structural viral proteins (VP1 to VP4, VP6, and VP7) and five or six nonstructural proteins (NSP1 to NSP5/6) [1]. A binary classification system is used to distinguish RVA based on the antigenic properties of the outer shell proteins, VP7 and VP4, that determine the G-genotype and P-genotype, respectively [1]. Furthermore, RVA can be separated into two main genogroups and one minor genogroup according to a whole-genome classification system, whereby a specific genotype is assigned to the 11 gene segments. These genogroups represent the genotype constellations that are present in most human strains globally [5,6]. Genogroup 1 (Wa-like) bears the constellation I1-R1-C1-M1-A1-N1-T1-E1-H1 and is often associated with the G genotypes G1, G3, G4, G9, and G12 and P genotype P[8]. Genogroup 2 (DS-1-like) includes G2P[4] strains and bears the constellation I2-R2-C2-M2-A2-N2-T2-E2-H2. Lastly, the minor genogroup 3 (AU-1-like) bears the I3-R3-C3-M3-A3-N3-T3-E3-H3 constellation and includes G3P[9] strains [7]. As of 5th May 2020, the Rotavirus Classification Working Group had identified at least 36 G, 51 P, 26 I, 22 R, 20 C, 20 M, 31 A, 22 N, 22 T, 27 E, and 22 H genotypes [8]. The whole-genome classification system has made it possible to analyse and understand the origin of various strains, interspecies transmission, and animal–human reassortment events [9]. Human Wa-like strains and porcine rotavirus strains share a common origin, whereas DS-1-like and AU-1-like strains have a common origin with bovine and feline strains, respectively [5]. 

In humans, G1-G4, G9, and G12 along with P[4], P[6], and P[8] are the most frequently detected, globally [10,11,12,13]. On the contrary, in porcine, predominant genotypes are G3-G5, G9, and G11 along with P[6], P[7], and P[13] [4,14]. Porcine rotaviruses bear the constellation I5-R1-C1-M1-A8-N1-T1/T7-E1-H1 [5,15,16,17,18,19,20]. While human Wa-like RVA differ from porcine rotaviruses in some gene segments (VP4, VP6, VP7, and NSP1), they both appear to have genotype 1 in the VP1, VP2, VP3, NSP2, NSP3, NSP4, and NSP5 gene segments. Hence, the suggestion that human Wa-like and porcine RVAs have arisen from a common ancestor [5]. 

The findings that show animals can serve as potential reservoirs for genetically diverse rotavirus strains that can be passed on to humans have elicited a large amount of interest and topics for further research [21]. Several novel and rare animal-like or animal–human reassortant rotavirus strains have been identified globally [22,23,24,25,26,27,28]. The detection of animal strains in humans is presumed to be as a result of zoonotic transmission, along with reassortment, which contributes to the diversity of circulating RVA [4,29,30]. Inter- and intragenogroup reassortment may occur when multiple RVA simultaneously infect a host. This is attributed to the segmented nature of the rotavirus genome [1,31]. It is, therefore, necessary to continuously carry out the monitoring of animal RVA and the role they play in contributing to the diversity of circulating RVA in humans.

The G5, one of the most common porcine genotypes, has sporadically been identified in human populations in Brazil (G5P[X]), Cameroon (G5P[7] and G5P[8]), Argentina (G5P[8]), and the United Kingdom(G5P[X]) [32,33,34,35,36]. The P[6] is presumed to be of porcine origin. They have also been identified in human populations [37,38,39,40]. The first human G5P[6] strain, LL36755, was detected in a child who had acute gastroenteritis in China in 2007 [41]. Other G5P[6] strains were detected in Vietnam, Taiwan, Bulgaria, Japan, and Thailand [37,42,43,44,45]. To date, the whole-genome of only two human G5P[6] strains—Bulgarian BG620 (nt sequences unavailable in the DDBJ, EMBL, and GenBank data libraries as of 13 August 2020) and Japanese Ryukyu-1120 (full open reading frame, available in GenBank)—have been analysed [45,46]. 

Diarrhoea is a burden for the Zambian healthcare system, with about 33% of the extreme cases being attributable to RVA [47,48,49]. In an attempt to generate disease burden attributable to rotavirus diarrhoea in children, the Zambian Ministry of Health, with support from WHO, launched rotavirus surveillance at the University Teaching Hospital (UTH) in 2006 [50,51]. Surveillance data generated provided evidence of the burden of rotavirus diarrhoea that supported the introduction of the rotavirus vaccine, Rotarix^®^, as a pilot project in Lusaka, Zambia in 2012, and was later rolled out nationwide in November 2013 [50]. According to the estimates reported by the World Health Organization (WHO) and the United Nations International Children’s Emergency Fund (WHO/UNICEF), rotavirus vaccine coverage in Zambia has been consistently high for the last six years, increasing from 73% in 2014 to 90% in 2019 [52]. Over this period, a sustained and significant reduction in rotavirus-associated hospitalisations and mortality was observed in children under 5 years [51]. 

The African Rotavirus Surveillance Network, coordinated by the World Health Organization Regional Office for Africa (WHO/AFRO), is actively monitoring the diversity and distribution of RVA genotypes in children hospitalised with acute diarrhoea [53]. Initially, the network was established with four countries in 2006, and expanded to 29 countries by the end of 2016 [54,55]. The Diarrhoeal Pathogens Research Unit at Sefako Makgatho University in Pretoria (South Africa) and the Noguchi Memorial Institute for Medical Research in Accra (Ghana) are the two WHO Rotavirus Regional Reference Laboratories (RRLs) for the network that conducts monitoring of rotavirus epidemiology in Africa [55]. The WHO/AFRO is currently supporting the University of the Free State-Next Generation Sequencing (UFS-NGS) unit to undertake rotavirus surveillance of rotavirus strains that circulated in Zambia between 2013 and 2016 at the whole-genome level. A G5P[6] strain, UFS-NGS-MRC-DPRU4723, was identified among these strains and was analysed so as to elucidate its origin and evolution. The sample was collected in 2014 from an unvaccinated 12 month old male hospitalised for gastroenteritis at Arthur Davison Children’s Hospital in Ndola, Zambia. 

## 2. Results

### 2.1. Nucleotide Sequencing and Identity of the Strain

Illumina^®^ MiSeq sequencing exhibited a phred score of Q30 and collectively yielded 98.8 Mbs of data for this specific sample. The whole genome of RVA/Human-wt/ZMB/UFS-NGS-MRC-DPRU4723/2014/G5P[6] was 18272 bps in size. The length and ORF of the 11 gene segments as determined by nucleotide sequencing are shown in Table 1. A BLASTn search was performed, and it appeared to exhibit maximum sequence identities of 95.7%–98.0% with porcine and human porcine-like strains (Table 1). Based on the whole genome classification system, RVA/Human-wt/ZMB/UFS-NGS-MRC-DPRU4723/2014/G5P[6] exhibited a G5-P[6]-I1-R1-C1-M1-A8-N1-T1-E1-H1 genotype constellation (Table 2). The genetic constellation of the study strain was compared to those of other G5 and non-G5 strains retrieved from the GenBank (Table 2).

### 2.2. Sequence and Phylogenetic Analysis

To investigate the potential origin of RVA/Human-wt/ZMB/UFS-NGS-MRC-DPRU4723/2014/G5P[6], phylogenetic trees were constructed for each of the 11 gene segments along with cognate gene sequences of RVA strains obtained from the GenBank. 

#### 2.2.1. Sequence and Phylogenetic Analysis of the VP7 Gene

**Figure 1:**
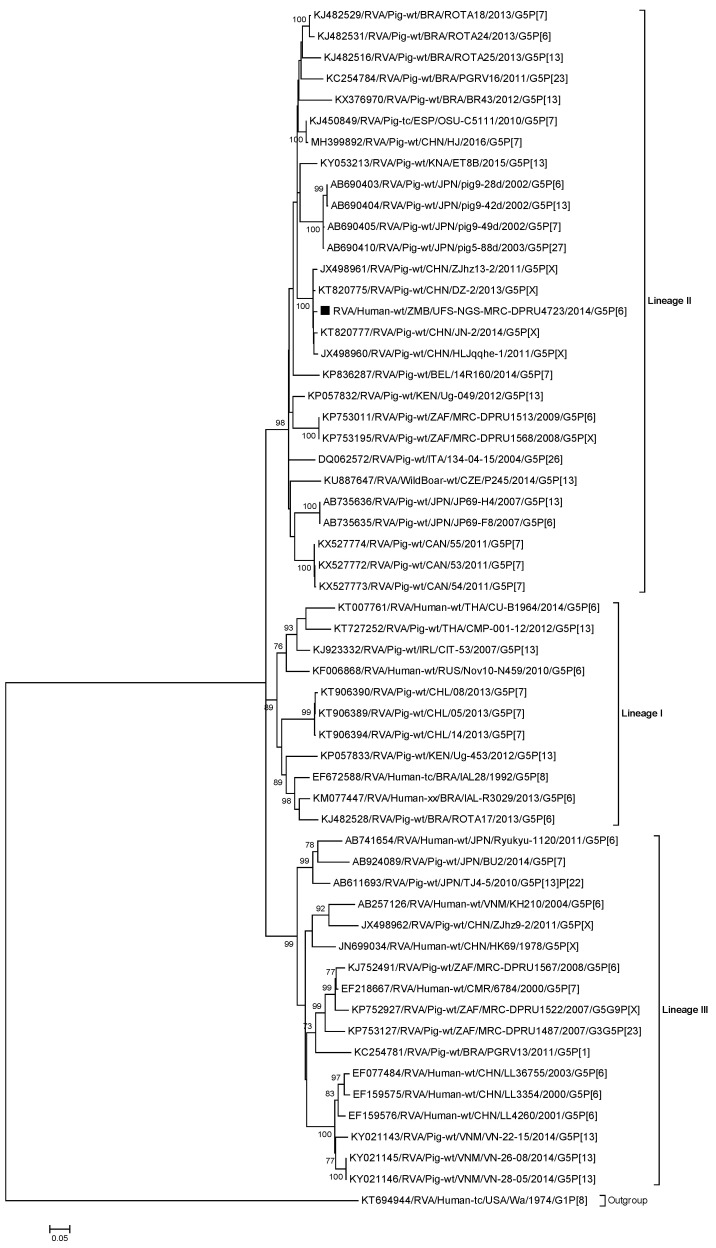
Phylogenetic tree constructed from the nucleotide sequences of the VP7 genes of strain RVA/Human-wt/ZMB/UFS-NGS-MRC-DPRU4723/2014/G5P[6] and representative strains. The position of strain RVA/Human-wt/ZMB/UFS-NGS-MRC-DPRU4723/2014/G5P[6] is shown by the black square (▪). Reference strains obtained from GenBank are represented by accession number, strain name, country, and year of isolation. The three closest strains, as identified by BLASTn, are also included. Bootstrap values ≥70% are shown adjacent to each branch node. Scale bar: 0.05 substitutions per nucleotide.

Phylogenetically, there are three known VP7 G5 lineages (I-III) [63]. The VP7 genes of RVA/Human-wt/ZMB/UFS-NGS-MRC-DPRU4723/2014/G5P[6] clustered into lineage II, which consisted only of porcine G5 strains from mainly Asia and the Americas (Figure 1). The VP7 gene showed the highest nucleotide (nt) and amino acid (aa) identities with the Chinese porcine strains RVA/Pig-wt/CHN/DZ-2/2013/G5P[X] nt (aa), 98.6% (99.0%), and RVA/Pig-wt/CHN/JN-2/2014/G5P[X] 98.5% (99.0%) and was distantly related to the strains within lineage III with lower sequence identities (nt, 83.4%–86.5%; aa, 90.4%–94.5%) (Figure 1; Supplementary data 1). Overall, strains within lineage II exhibited sequence identities that were in the range nt, 89.6%–98.6%; aa, 92.4%–99.0% (Supplementary data 1). 

The comparison of the amino acid sequence of RVA/Human-wt/ZMB/UFS-NGS-MRC-DPRU4723/2014/G5P[6] to reference G5 strains e.g., RVA/Pig-wt/THA/CMP-001-12/2012/G5P[13] (lineage I), RVA/Pig-wt/BRA/ROTA24/2013/G5P [6] (lineage II) and RVA/Human-wt/JPN/Ryukyu-1120/2011/G5P[6] (lineage III) within each of the three lineages revealed a high identity (range 90.0%–94.9% (Supplementary data 1; Supplementary data 2a). Numerous substitutions were identified in the nine VP7 variable regions, VR-1 to VR-9 [64]: VR-1 (I9V and I19V), VR-2 (V27T and V29T), VR-3 M/F39L, I40V, V41I, L/I43V, I/L/V47F, R49K, and A50T), VR-4 (K/A65T, V/M68A, M/A72T, and M/Q75T), VR-5/antigenic site A (N/S/D/T96A), VR-6 (I129V and D130E), VR-7/antigenic site B (N145D and A/V/E146G), VR-8/antigenic site C (L/S208T, A210T, T/V212I, S/A213I, I/M217T, V218I, and S220N), and VR-9/antigenic site F (A/M241T and S242N). 

#### 2.2.2. Sequence and Phylogenetic Analysis of the VP4 Gene

**Figure 2:**
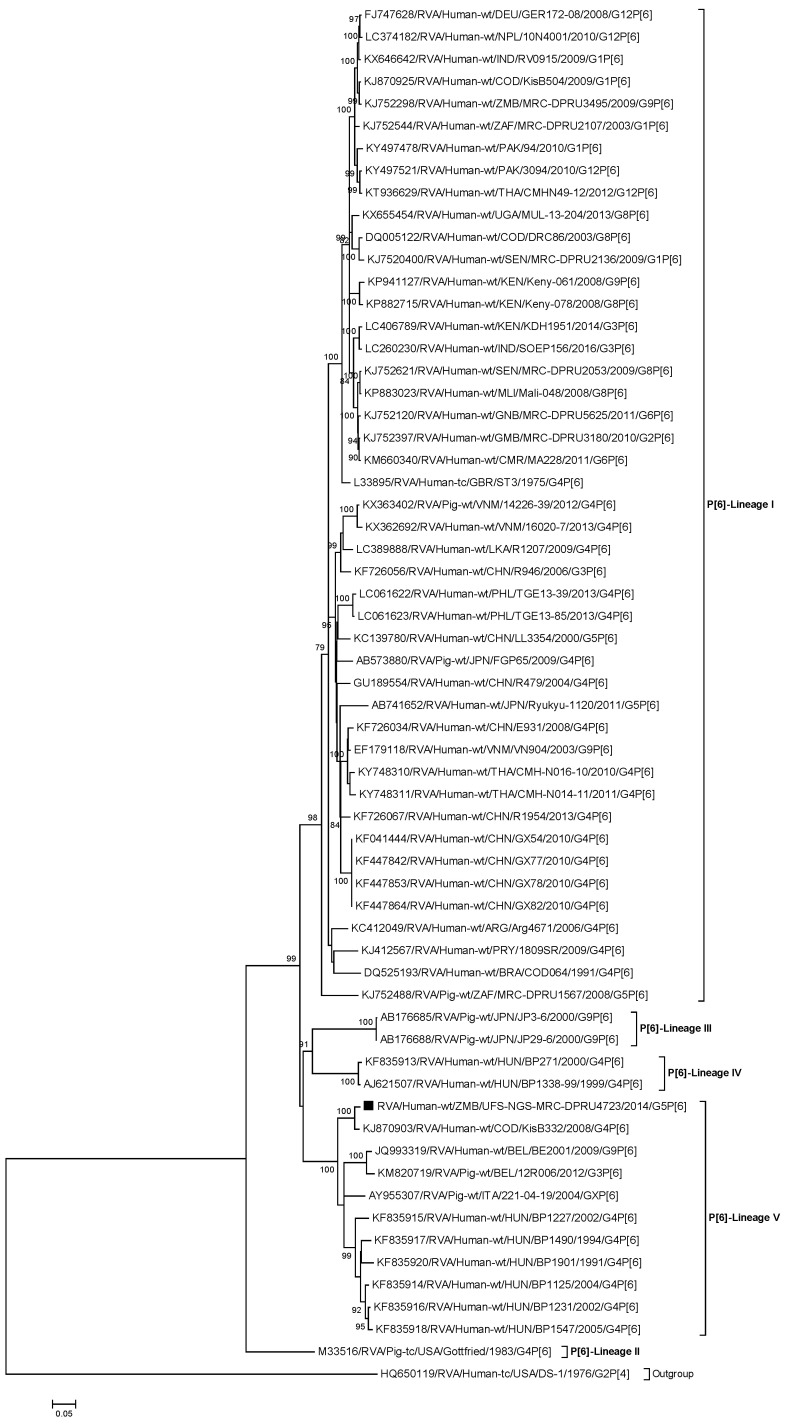
Phylogenetic tree constructed from the nucleotide sequences of the VP4 genes of strain RVA/Human-wt/ZMB/UFS-NGS-MRC-DPRU4723/2014/G5P[6] and representative strains. The position of strain RVA/Human-wt/ZMB/UFS-NGS-MRC-DPRU4723/2014/G5P[6] is shown by the black square (▪). Reference strains obtained from GenBank are represented by accession number, strain name, country, and year of isolation. The three closest strains, as identified by BLASTn, are also included. Bootstrap values ≥70% are shown adjacent to each branch node. Scale bar: 0.05 substitutions per nucleotide.

The VP4 gene of RVA/Human-wt/ZMB/UFS-NGS-MRC-DPRU4723/2014/G5P[6] was phylogenetically compared to the already established five lineages (I-V) of genotype P[6] [65] (Figure 2). The P[6] gene of the study strain clustered into lineage V, which consisted of porcine and putative human porcine-like strains detected in parts of Europe and one African strain. A similarity analysis of the P[6] gene of the study strain with strains obtained from GenBank showed that the Zambian G5P[6] exhibited the highest sequence identity of 98.1% (98.3%) with a porcine-like human strain RVA/Human-wt/COD/KisB332/2008/G4P[6] from the Democratic Republic of Congo (Supplementary data 1). All the African strains clustered into a separate lineage, lineage I, with sequence identities of 85.7%–86.8% (92.5%–93.9%) (Supplementary data 1). 

The deduced amino acid sequences of the VP4 gene of RVA/Human-wt/ZMB/UFS-NGS-MRC-DPRU4723/2014/G5P[6] along with the reference P[6] strain from each of the five lineages was compared (Supplementary data 2b). The reference strains shared high amino acid identities ranging from 91.0% to 98.3% (Supplementary data 1). Several amino acid changes were identified throughout the VP4 protein, and most of the substitutions were concentrated in the hypervariable region (amino acid 71-208) which houses the VR-3 (92%–192) and includes a neutralization site at amino acid 135 [66,67]. Several amino acid substitutions were observed among the P[6] lineage I strains [65] at the VR-3 (L105I, V108I and T134S) and VR-8 (D602N) variable regions. Other amino acid substitutions were identified among the P[6] lineages at VR-1 (S30N), VR-2 (I61V), VR-3 (V112I, N114S, V130I, H182N and T189S), VR-4 (I280V), and VR-9 (E698K). The potential trypsin cleavage sites at residues 241 and 247 [68] were highly conserved in all the strains with three substitutions at positions 242 (I to V), 243 (A to T), and 244 (H to Y). 

#### 2.2.3. Phylogenetic Analysis of the VP6 Gene

**Figure 3:**
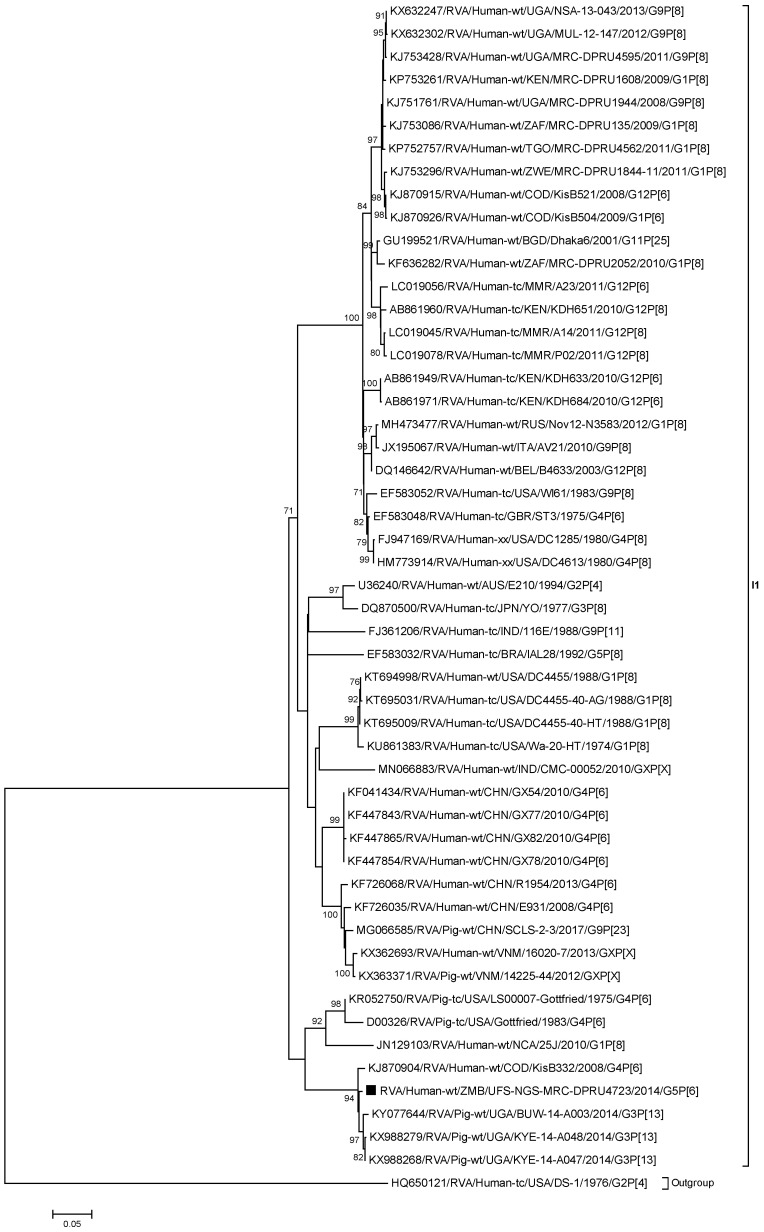
Phylogenetic tree constructed from the nucleotide sequences of the VP6 genes of strain RVA/Human-wt/ZMB/UFS-NGS-MRC-DPRU4723/2014/G5P[6] and representative strains. The position of strain RVA/Human-wt/ZMB/UFS-NGS-MRC-DPRU4723/2014/G5P[6] is shown by the black square (▪). Reference strains obtained from GenBank are represented by accession number, strain name, country, and year of isolation. The three closest strains, as identified by BLASTn, are also included. Bootstrap values ≥70% are shown adjacent to each branch node. Scale bar: 0.05 substitutions per nucleotide.

The VP6 gene of RVA/Human-wt/ZMB/UFS-NGS-MRC-DPRU4723/2014/G5P[6] clustered closely with divergent African porcine strains from Uganda (RVA/Pig-wt/UGA/BUW-14-A003/2014/G3P[13], RVA/Pig-wt/UGA/KYE-14-A048/2014/G3P[13], and RVA/Pig-wt/UGA/KYE-14-A047/2014/G3P[13]) and a human porcine-like strain from the Democratic Republic of Congo (RVA/Human-wt/COD/KisB332/2008/G4P[6]) which displayed nt(aa) sequence identities ranging from 98.6% to 98.9% (98.9%–99.7%) (Figure 3, Supplementary data 1). Porcine-like Asian strains such as RVA/Human-wt/CHN/GX54/2010/G4P[6] and RVA/Human-wt/CHN/E931/2008/G4P[6] clustered separately, displaying identities of 88.7%–90.2% (97.5%–98.7%) (Supplementary data 1). 

#### 2.2.4. Phylogenetic Analysis of VP1 Gene

The VP1 gene of RVA/Human-wt/ZMB/UFS-NGS-MRC-DPRU4723/2014/G5P[6] clustered only with porcine and porcine-like human strains from Asia (China and Vietnam) (Supplementary data 3a). The VP1 gene exhibited a maximum nt (aa) sequence identity of 96.8% (98.9%) with the Chinese human porcine-like reassortant strains RVA/Human-wt/CHN/GX82/2010/G4P[6], RVA/Human-wt/CHN/GX78/2010/G4P[6], RVA/Human-wt/CHN/GX77/2010/G4P[6], and RVA/Human-wt/CHN/GX54/2010/G4P[6] (Supplementary data 1). Overall, the Asian strains within the cluster showed sequence identities of 94.1%–96.8% (97.9%–98.9%). Human non-porcine African strains clustered separately, with lower identities of 88.2%–88.8% (96.3%–97.3%) (Supplementary data 1). 

#### 2.2.5. Phylogenetic Analysis of VP2 Gene

The VP2 gene of strain RVA/Human-wt/ZMB/UFS-NGS-MRC-DPRU4723/2014/G5P[6] fell into a distinct cluster predominantly composed of porcine and porcine-like human strains from Asia (China, India, Vietnam, South Korea, and Sri Lanka) (Supplementary data 3b). The VP2 gene of the study strain showed a maximum nt (aa) sequence identity of 96.6% (90.9%) with a Sri Lankan porcine-like human strain RVA/Human-wt/LKA/R1207/2009/G4P[6] (Supplementary data 1).

#### 2.2.6. Phylogenetic Analysis of VP3 Gene

The VP3 gene of strain RVA/Human-wt/ZMB/UFS-NGS-MRC-DPRU4723/2014/G5P[6] clustered in a lineage composed mainly of Asian (Asia and Thailand) porcine and porcine-like human strains (Supplementary data 3c), and exhibited the highest nt (aa) sequence identity with the Chinese porcine-like human strains—RVA/Human-wt/CHN/R946/2006/G3P[6], 95.8% (97.8%) and RVA/Human-wt/CHN/E931/2008/G4P[6], 95.7% (98.0%) (Supplementary data 1). The overall similarities of the Asian strains within the lineage ranged from 84.8% to 95.8% (92.7%–97.8%) (Supplementary data 1). Non-porcine African strains clustered separately and showed lower sequence identities of 84.1%–84.5% (92.1%–92.7%) (Supplementary data 1). 

#### 2.2.7. Phylogenetic Analysis of NSP1 Gene

The NSP1 gene of strain RVA/Human-wt/ZMB/UFS-NSG-MRC-DPRU4723/2014/G5P[6] was assigned to a porcine genotype A8 and clustered among Asian (Vietnam, China, and Bangladesh) porcine and porcine-like human strains and an African (Ghana) porcine strain (Supplementary data 3d). The NSP1 gene of the study strain was closest to strain RVA/Human-tc/VNM/NT0042/2007/G4P[6] displaying a nt(aa) sequence identity of 98.2% (97.9%) (Supplementary data 1). The porcine and porcine-like human strains from Europe and the Americas clustered separately showing sequence identities of 84.2%–85.9% (85.4%–88.2%) and 84.1%–85.9% (83.7%–88.3%), respectively (Supplementary data 1). 

#### 2.2.8. Phylogenetic Analysis of NSP2 Gene

The NSP2 gene of strain RVA/Human-wt/ZMB/UFS-NGS-MRC-DPRU4723/2014/G5P[6] clustered with Asian and European porcine and porcine-like human strains (Supplementary data 3e). The Nt(aa) similarity analysis showed that the NSP2 gene of the study strain was most similar to the Chinese porcine strains RVA/Pig-wt/CHN/YN/2012/GXP[X] and RVA/Pig-tc/CHN/SCMY-A3/2017/G9P[23]—96.8% (97.8%) (Supplementary data 1). Two African porcine strains, RVA/Pig-wt/ZAF/MRC-DPRU1487/2007/G3G5P[23] and RVA/Pig-wt/ZAF/MRC-DPRU1557/2008/G4G5P[23], were seen to cluster within the same lineage with sequence identities of 93.6%–93.7% (97.5%–97.8%) (Supplementary data 1). 

#### 2.2.9. Phylogenetic Analysis of NSP3 Gene

The NSP3 gene of strain RVA/Human-wt/ZMB/UFS-NGS-MRC-DPRU4723/2014/G5P[6] clustered closely with porcine and porcine-like human strains mainly from Asia (Thailand and Vietnam) and exhibited a maximum nt(aa) sequence identities of 96.5%–97.0% (98.4%–98.7%) with the strains RVA/Human-wt/VNM/30378/2009/G26P[19], RVA/Pig-wt/VNM/12070-4/2012/GXP[X], RVA/Human-wt/VNM/NT0205/2007/G4P[6], and RVA/Human-wt/VNM/NT0621/2008/G4P[6] (Supplementary data 1; Supplementary data 3f).

#### 2.2.10. Phylogenetic Analysis of NSP4 Gene

The NSP4 gene of strain RVA/Human-wt/ZMB/UFS-NGS-MRC-DPRU4723/2014/G5P[6] clustered with porcine and porcine-like human strains identified in Asia (China and Vietnam) and a porcine-like human strain from the Americas (Brazil) (Supplementary data 3g). In this cluster, the closest strains to UFS-NGS-MRC-DPRU4723 were the wild pig strains (RVA/WildBoar-wt/CZE/P828/2015/G9P[23] and RVA/WildBoar-wt/CZE/P830/2015/G9P[23]) from the Czech Republic, with nt(aa) sequence identities of 97.5% (98.3%) (Supplementary data 1). The Asian strains within the cluster showed nt(aa) similarities of 96.2%–97.3% (97.7%–98.9%). Porcine and porcine-like human strains from the Americas clustered separately and exhibited identities of 87.2%–96.4% (94.3%–98.9%) (Supplementary data 1). 

#### 2.2.11. Phylogenetic Analysis of the NSP5 Gene

The NSP5 gene of strain RVA/Human-wt/ZMB/UFS-NGS-MRC-DPRU4723/2014/G5P[6] clustered with porcine strains from Asia and showed the highest nt(aa) sequence identity of 98.6% (100%) with the porcine strains RVA/Pig-wt/CHN/TM-a/2009/G3P[8] and RVA/Pig-tc/CHN/TM-a-P20/2018/G9P[23] identified in China (Supplementary data 1; Supplementary data 3h). Overall, the porcine and porcine-like human strains from Asia and the Americas displayed nt(aa) identities of in the range 94.8%–98.6% (98.0%–100%) and 93.9%–96.1% (95.9%–99.0%), respectively (Supplementary data 1).

### 2.3. Reassortment Analysis 

**Figure 4:**
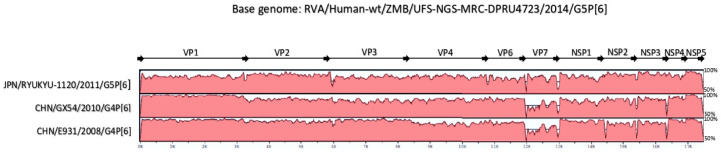
mVISTA whole genome nucleotide alignment comparing the Zambian G5P[6] strain (RVA/Human-wt/ZMB/UFS-NGS-MRC-DPRU4723/2014G5P[6]) with the G5P[6] strain from Japan (Ryukyu-1120), whose whole genome sequence had been determined, and with selected porcine-like human P[6] strains from China (GX54 and E931). Strain names are shown on the left, and the proteins VP1-VP4, VP6-VP7, and NSP1-NSP5 are indicated on the top. The bottom scale indicates distance in kb. Percentile values on the right indicate sequence-based similarity between the study strain and the respective reference strains. Shading indicates the level of conservation.

The concatenated whole genome alignment of RVA/Human-wt/ZMB/UFS-NGS-MRC-DPRU4723/2014/G5P[6], together with the Japanese G5P[6] strain and selected Chinese porcine-like human P[6] strains, was visualised (Figure 4). The whole genome of the Zambian G5P[6] strain demonstrated a relatively high degree of conservation with the Japanese G5P[6] strain and the two Chinese G4P[6] strains. With the exception of VP7 and VP4, the genome of the Chinese strain E931 exhibited the overall highest genomic conservation to the study strain. With the exception of VP7, VP3, and NSP1 genes, the Chinese strain GX54 shared a highly conserved genome with the study strain. The Japanese strain Ryukyu-1120 demonstrated a highly similar genome to the study strain for seven of the 11 genes, the exceptions being VP1, VP3, VP6, and VP7. The results of this analysis confirmed the genetic similarity between RVA/Human-wt/ZMB/UFS-NGS-MRC-DPRU4723/2014/G5P[6] and Asian (Chinese) porcine-like human strains, hence suggesting that the Zambian G5P[6] strain may have been derived via reassortment events.

## 3. Discussion

The detection of genotype G5 in humans, which is typical for pigs, is possibly due to interspecies transmission [35,45]. In Zambia, as with many countries in Africa, humans and farm animals live in proximity. The interaction between humans and animals could be the primary cause for zoonotic transmission, which could result in genetic reassortments and perhaps other mechanisms of genetic diversity, ultimately leading to the introduction and spread of animal genotypes into human populations [69].

In this study, an analysis was conducted on a sample collected from a child admitted to a paediatric ward presenting with clinical symptoms (vomiting, diarrhoea, and fever) that are usually present during typical rotavirus infection. This raises the question whether such animal-derived strains are capable of mutating and effectively spreading within/across human populations as in the case of established typical Wa-like and DS-1-like genotype constellations, with the same magnitude of rotavirus disease severity. Furthermore, taking into consideration that the G5 and P[6] genotypes are not included in the currently available vaccines, the probability for such strains to have the potential to spread more swiftly from human to human may have implications for the effectiveness of current rotavirus vaccine candidates that are in use in African countries.

This study identified the complete genome of a reassortant porcine-like human strain, G5P[6], that showed the genotype constellation G5-P[6]-I1-R1-C1-M1-A8-N1-T1-E1-H1, which is commonly found in porcine and porcine-like human rotavirus strains [19]. RVA/Human-wt/ZMB/UFS-NGS-MRC-DPRU4723/2014/G5P[6] was found to share the same constellation (I1-R1-C1-M1-A8-N1-T1-E1-H1) with the archival porcine strain, Gottfried, and porcine-like human strains—BG260, E931, and GX54 [5,46,56,58]. In addition, porcine strains 12R002, 12R005, and 12R006, as well as porcine-like human strains Ryukyu-1120, mani-97, 30378, rj24598, and BE2001 shared the same constellation with strain RVA/Human-wt/ZMB/UFS-NGS-MRC-DPRU4723/2014/G5P[6] with the exception of VP6 (I5 instead of I1) and NSP3 (T7 instead of T1 gene segments) [20,25,26,45,70]. 

A phylogenetic analysis of RVA/Human-wt/ZMB/UFS-NGS-MRC-DPRU4723/2014/G5P[6] showed that this strain was a possible reassortant, as it was closely related to both porcine and porcine-like human strains, predominantly from Asia, than to typical human RVA strains. The VP6, VP7, NSP2, NSP4, and NSP5 segments of this strain showed a close similarity to porcine strains. Although the remaining gene segments (VP1, VP3, VP4, and NSP3) were closely related to human strains, all of these were porcine-like human strains [26,56,58,59,60,70]. With a genotype 1 (Wa-like) backbone, this finding is consistent with the hypothesis that human Wa-like strains and porcine strains have a common ancestor [5]. However, the origin of the VP2 gene of the study strain was not very definitive, as it was not only close to porcine and porcine-like human strains but also to three human strains (DC1476, DC582, and DC1127). Phylogenetically, the clusters of these three strains were shown to be distinctive from the genes of contemporary, wild-type human strains [71]. Notably, the VP7 gene of RVA/Human-wt/ZMB/UFS-NGS-MRC-DPRU4723/2014/G5P[6] was located in lineage II, which comprised only porcine strains, hence implying the possibility of porcine-to-human interspecies transmission [63]. Phylogenetic analysis of porcine and human P[6] strains indicated that both porcine and human P[6] strains were present in P[6] lineages I, III, and V, hence showing that human P[6] strains might have separately emerged from at least three porcine-to-human transmissions [65]. This finding supports the Zambian G5P[6] strain, as the VP4 gene clustered and shared high nucleotide and amino acid identities with lineage V of P[6] porcine and porcine-like human strains. The NSP1 gene was most similar to porcine-like human strains. However, it was revealed to have the porcine genotype A8. Taking this together, it is likely that RVA/Human-wt/ZMB/UFS-NGS-MRC-DPRU4723/2014/G5P[6] originated by zoonotic transmission, coupled with reassortment events.

Several amino acid changes were identified in the nine variable regions when the VP7 gene of the study strain was compared to other G5 strains within each of the three lineages [64]. Additionally, the previously described conserved N-glycosylation site at residues 69–71 within the variable region 4 (VR-4) was found to be conserved in all the G5 strains used in this analysis [64,72]. Four major antigenic regions have been described for the VP7 protein in rotaviruses (A, B, C and F) [73,74]. Marked differences in the antigenic regions of RVA/Human-wt/ZMB/UFS-NGS-MRC-DPRU4723/2014/G5P[6] were seen when it was compared to other globally circulating G5 strains. Usually, antigenic regions A and C are said to be conserved within serotypes [75]. However, multiple substitutions were observed in these regions when comparing the Zambian G5 strain to other G5 strains globally.

The amino acid sequence for the VP4 gene was 775 amino acids long and displayed amino acid identity values ranging from 91.0% to 98.3% with the reference P[6] strains. Considering it has been established that strains with amino acid identities greater than 89% belong to the same P genotype [76], our findings show that RVA/Human-wt/ZMB/UFS-NGS-MRC-DPRU4723/2014/G5P[6] belongs to the genotype P[6]. The analysis of the amino acid sequences showed that the hypervariable region (amino acid 71-208) which houses the variable region 3 (VR-3) contained most of the substitutions. Furthermore, the potential trypsin cleavage sites [68] were conserved in all the P[6] strains. Several amino acid substitutions were observed among the lineage I P[6] strains. The presence of several amino acid changes in the VP4 gene of this strain compared to other circulating P[6] strains globally is in agreement with the hypothesis that the P[6] gene has been introduced to humans via independent reassortment events [40,65,77]. 

Rotaviruses are genetically diverse in nature and are host-species specific, suggesting that host species barriers and restrictions exist. However, rotaviruses of animal origin may cross the host species barrier and may acquire human rotavirus gene segments, which enables the viruses to efficiently spread across human populations [4]. In this regard, G5 rotavirus strains have sporadically been documented in Latin America, Asia, Europe, and Africa [33,34,35,36,37,41,45,46]. Porcine P[6] strains seem to pose a lesser species barrier to humans [20]. Even though the relationship between porcine and human rotaviruses has already been established [5], whole genome analysis in this study presented the possible occurrence of interspecies transmission and reassortment between human and porcine rotaviruses. 

## 4. Materials and Methods 

### 4.1. Ethics Statement

This is a subset of a major project which involved the whole genome characterisation of 133 specimens collected in Zambia from 2013 through 2016 as part of the surveillance supported by the WHO/AFRO (reference 2017/757922-0) in collaboration with the University of the Free State (UFS-NGS). Ethical clearance for the main project was obtained under ethics number HSREC130/2016(UFS-HSD2016/1082) from the Health Science Research Ethics Committee (HSREC), University of the Free State, Bloemfontein, South Africa. Furthermore, this specific study was approved by the HSREC under ethics number UFS-HSD2020/0277/2104. 

### 4.2. Sample Collection

The sample was collected in 2014 from an unvaccinated 12 month old male at Arthur Davidson Children’s Hospital (ADCH) in Ndola, a rotavirus surveillance sentinel site. The child had travelled with parents from Kasama, a town in the Northern Province of Zambia which is approximately 760 km away from Ndola, Zambia. This child was admitted to a paediatric ward at ADCH, with gastroenteritis of four days duration and a history of fever. Frequency of vomiting and diarrhoea was three episodes and two episodes, respectively, in the previous 24 h. The level of dehydration was assessed as mild and the child received an oral rehydration solution and was discharged after a few days. The stool sample was screened using the enzyme immunoassay (EIA) technique for the presence of RVA antigen in the Virology laboratory in Lusaka. It was randomly picked and sent to the Diarrhoeal Pathogens Research Unit (DPRU), a World Health Organization Rotavirus Regional Reference Laboratory (WHO-RRL) in Pretoria, South Africa, as part of the WHO/AFRO annual rotavirus surveillance. Conventional genotyping was carried out at DPRU. Thereafter, the sample was shipped to the UFS-NGS unit for sequencing and whole-genome analysis.

### 4.3. Viral dsRNA Extraction

The viral double-stranded RNA (dsRNA) was extracted from human stool suspensions using a previously described method with modifications [78]. Approximately 100 mg stool was suspended in 200 µL phosphate-buffered saline (PBS) solution (Sigma-Aldrich^®^, St Louis, MO, United States). The faecal suspension was mixed with 900 µL TRI Reagent^®^ LS (Molecular Research Centre, Cincinnati, OH, United States) and homogenized for five minutes. A 300 µL volume of chloroform (Sigma-Aldrich^®^, St Louis, MO, United States) was used to achieve phase separation, which was followed by centrifugation (Eppendorf microcentrifuge 5427 R, Germany) at 17,319× *g* for 20 min at 4 °C. The supernatant was precipitated using 700 µL ice-cold isopropanol (Sigma-Aldrich^®^, United States) and centrifuged (Eppendorf microcentrifuge 5427 R, Germany) at 17,319× *g* for 30 min at 4 °C. The supernatant was discarded, and the tubes were air-dried for 5 min, followed by the precipitation of single-stranded RNA (ssRNA) using 30 µL 8 M lithium chloride (Sigma, St Louis, MO, United States) at 4 °C for 16 h. The dsRNA was purified using the MinElute gel extraction kit (Qiagen, Hilden, Germany). RNA integrity was determined by electrophoresis on 1% TBE agarose gel stained with ethidium bromide (Sigma-Aldrich^®^, St Louis, MO, United States), which was visualised on a G: Box UV transilluminator (Syngene, Cambridge, United Kingdom). 

### 4.4. cDNA Synthesis and Purification

cDNA synthesis was carried out using the Maxima H Minus Double-stranded cDNA kit (Thermo Fisher Scientific, Waltham, MA, United States) according to the manufacturer’s instructions with minor modifications captured at the UFS-NGS SOP, whereby the dsRNA was denatured at 95 °C for 5 min. First strand synthesis was carried out for two hours at 50 °C. Random hexamer primer was employed for cDNA synthesis. The cDNA was purified using the MSB^®^ Spin PCRapace purification kit (Stratec, Invitek Molecular, Berlin, Germany). 

### 4.5. DNA Library Preparation and Illumina^®^ MiSeq Sequencing

DNA libraries for Illumina^®^ sequencing were prepared using the Nextera^®^ XT DNA library preparation kit (Illumina, San Diego, CA, United States) according to the manufacturer’s instructions. Briefly, DNA was tagmented at 55 °C for five minutes followed by ligation to Illumina^®^ sequencing index 1 and index 2 adapters by PCR amplification. Size selection and clean-up of the DNA libraries was performed using Agencourt AMPure XP beads (Beckman Coulter, South Kraemer Boulevard Brea, CA, United States). The quantity of DNA was determined on the Qubit 2.0 fluorimeter (Invitrogen, Carlsbad, CA, United States), and a quality check of the libraries was performed on a Bioanalyzer 2100 (Agilent Technologies, Santa Clara, CA, United States). After this, sequencing was performed on an Illumina^®^ MiSeq sequencer (Illumina, San Diego, CA, United States) using a MiSeq reagent kit v3 for 600 cycles (2 × 300 bp paired reads) with a 10% PhiX DNA control spike-in.

### 4.6. Genome Assembly

The raw reads obtained in FASTQ format were assembled using Geneious Prime^®^ 2019.2.1 (https://www.geneious.com/; [79]). Briefly, the paired-end reads were merged into single reads and trimmed to remove low quality and short reads. The reads were mapped to reference sequences obtained from GenBank. Consensus sequences covering the complete open reading frame (ORF) were submitted to the National Centre for Biotechnology Information (NCBI) GenBank and assigned accession numbers MT271025–MT271035. The ORF lengths were 3267 (VP1), 2673 (VP2), 2508 (VP3), 2328 (VP4), 1194 (VP6), 981 (VP7), 1482 (NSP1), 954 (NSP2), 942 (NSP3), 528 (NSP4), and 594 (NSP5).

### 4.7. Assignment of Genotypes

The genotypes of each of the 11 rotavirus genome segments were determined using the online Virus Pathogen Resource (ViPR). 

### 4.8. Phylogenetic Analysis

Gene-specific multiple sequence alignments were made using the MAFFT plugin implemented in Geneious Prime^®^ 2019.2.1 and the MUSCLE algorithm embedded in MEGA 6.06 (for the VP2 and NSP1 segments) [80,81]. Once aligned, the DNA Model Test program in MEGA 6.06 was used to identify the optimal evolutionary model for each genome segment [82]. Using an Akaike information criterion (corrected) (AICc), the following models were found to best fit the data: HKY+G+I (VP1), GTR+G+I (VP2, VP3, and VP4), T92+G (VP6, NSP1, NSP2, NSP3, NSP4, and NSP5), and T92+G+I (VP7). Maximum likelihood trees were constructed using the optimal models in MEGA version 6.06 [82,83] with 1000 bootstrap replicates to estimate branch support [84]. The shared nucleotide and amino acid sequence identities among strains were calculated for each gene using the *p*-distance algorithm in MEGA 6.06. Analysis and visualization of the aligned concatenated whole genomes was performed on the mVISTA online platform [85]. 

## 5. Conclusions

In summary, RVA/Human-wt/ZMB/UFS-NGS-MRC-DPRU4723/2014/G5P[6] was a reassortant possessing gene segment of porcine and porcine-like human origin, and was closest to Asian strains. It is presumed that pigs play a crucial part as a source for new or newly-evolved emerging human rotaviruses. This highlights the need for continuous large-scale surveillance and whole genome analysis of circulating porcine and human rotaviruses. Furthermore, it was imperative to examine the prevalence of G5P[6] strains in Zambia. Eventually, this should result in a greater understanding of the genes that determine the transmission between hosts successfully as well as to gain insights on complex reassortment patterns between porcine and human rotaviruses.

## Figures and Tables

**Table 1:** The segment and ORF lengths of strain UFS-NGS-MRC-DPRU4723 and the highest sequence identities obtained using the Basic Local Alignment Search Tool (BLAST).

GENOME SEGMENT Encoding	GenBank Accession no.	Segment Length	ORF Length	Results of Blast Search			
				Most Similar Strain	GenBank Accession no.	Similarity (%)	Reference
**VP1**	MT271025	3302	3267	GX54	KF041441	96.7	[56]
**VP2**	MT271026	2673	2673	R1207	LC389886	96.5	[57]
**VP3**	MT271027	2591	2508	R946	KF726060	95.7	[58]
**VP4**	MT271028	2359	2328	KisB332	KJ870903	98.0	[59]
**NSP1**	MT271029	1512	1482	NT0042	LC095894	98.1	[60]
**VP6**	MT271030	1356	1194	KYE-14-A048	KX988279	98.7	[29]
**NSP3**	MT271031	1076	942	12070-4	KX363287	97.1	[61]
**NSP2**	MT271032	954	954	YN	KJ466987	96.8	[https://www.ncbi.nlm.nih.gov/nuccore/KJ466987]
**VP7**	MT271033	1054	981	JN-2	KT820777	98.0	[https://www.ncbi.nlm.nih.gov/nuccore/KT820777]
**NSP4**	MT271034	751	528	14150-54	KX363354	97.7	[61]
**NSP5**	MT271035	644	594	R479	GU189559	97.6	[62]

**Table 2:** Genotype natures of the 11 gene segments of Zambian strain UFS-NGS-MRC-DPRU4723 compared with those of selected human and porcine strains.

Strain	Genotype										
	VP7	VP4	VP6	VP1	VP2	VP3	NSP1	NSP2	NSP3	NSP4	NSP5
**RVA/Human-wt/ZMB/UFS-NGS-MRC-DPRU4723/2014/G5P[6]**	**G5**	**P[6]**	I1	R1	C1	M1	**A8**	N1	T1	E1	H1
RVA/Human-wt/BGR/BG260/2008/G5P[6] *	**G5**	**P[6]**	I1	R1	C1	M1	**A8**	N1	T1	E1	H1
RVA/Human-wt/JPN/Ryukyu-1120/2011/G5P[6]	**G5**	**P[6]**	**I5**	R1	C1	M1	**A8**	N1	T1	E1	H1
RVA/Human-wt/CHN/LL3354/2000/G5P[6]	**G5**	**P[6]**	**I5**	-	-	-	-	-	-	E1	-
RVA/Human-wt/CHN/LL4260/2001/G5P[6]	**G5**	**P[6]**	-	-	-	-	-	-	-	E1	-
RVA/Human-wt/CHN/LL36755/2003/G5P[6]	**G5**	**P[6]**	-	-	-	-	-	-	-	E1	-
RVA/Human-wt/VNM/KH210/2004/G5P[6]	**G5**	**P[6]**	-	-	-	-	-	-	-	E1	-
RVA/Human-wt/TWN/03-98P50/2009/G5P[6] *	**G5**	**P[6]**	**I5**	-	-	-	-	-	-	E1	-
RVA/Human-wt/CMR/6784/ARN/2000/G5P[7]	**G5**	**P[7]**	**I5**	R1	C1	M1	A1	N1	T1	E1	H1
RVA/Human-tc/BRA/IAL28/1992/G5P[8]	**G5**	P[8]	**I5**	R1	C1	M1	A1	N1	T1	E1	H1
RVA/Pig-tc/USA/OSU/1975/G5P[7]	**G5**	**P[7]**	**I5**	R1	C1	M1	A1	N1	T1	E1	H1
RVA/Pig-wt/BEL/12R002/2012/G5P[7]	**G5**	**P[7]**	**I5**	R1	C1	M1	**A8**	N1	**T7**	E1	H1
RVA/Pig-wt/JPN/BU2/2014/G5P[7]	**G5**	**P[7]**	**I5**	R1	C1	M1	**A8**	N1	T1	E1	H1
RVA/Human-tc/USA/Wa/1974/G1P[8]	G1	P[8]	I1	R1	C1	M1	A1	N1	T1	E1	H1
RVA/Human-tc/USA/DS-1/1976/G2P[4]	G2	P[4]	I2	R2	C2	M2	A2	N2	T2	E2	H2
RVA/Human-tc/JPN/AU-1/1982/G3P[9]	**G3**	P[9]	I3	R3	C3	M3	A3	N3	T3	E3	H3
RVA/Pig-wt/BEL/12R006/2012/G3P[6]	**G3**	**P[6]**	**I5**	R1	C1	M1	**A8**	N1	T1	E1	H1
RVA/Human-tc/GBR/ST3/1974/G4P[6]	**G4**	**P[6]**	I1	R1	C1	M1	A1	N1	T1	E1	H1
RVA/Pig-tc/USA/Gottfried/1975/G4P[6]	**G4**	**P[6]**	I1	R1	C1	M1	**A8**	N1	T1	E1	H1
RVA/Human-tc/CHN/R479/2004/G4P[6]	**G4**	**P[6]**	**I5**	R1	C1	M1	A1	N1	**T7**	E1	H1
RVA/Human-wt/CHN/E931/2008/G4P[6]	**G4**	**P[6]**	I1	R1	C1	M1	**A8**	N1	T1	E1	H1
RVA/Human-wt/COD/KisB332/2008/G4P[6]	**G4**	**P[6]**	I1	R1	C1	M1	A1	N1	**T7**	E1	H1
RVA/Human-wt/CHN/GX54/2010/G4P[6]	**G4**	**P[6]**	I1	R1	C1	M1	**A8**	N1	T1	E1	H1
RVA/Pig-wt/BEL/12R005/2012/G4P[7]	**G4**	**P[7]**	**I5**	R1	C1	M1	**A8**	N1	**T7**	E1	H1
RVA/Human-wt/BEL/BE2001/2009/G9P[6]	**G9**	**P[6]**	**I5**	R1	C1	M1	**A8**	N1	**T7**	E1	H1
RVA/Human-tc/USA/WI61/1983/G9P[8]	**G9**	P[8]	I1	R1	C1	M1	A1	N1	T1	E1	H1
RVA/Human-wt/BEL/B3458/2003/G9P[8]	**G9**	P[8]	I1	R1	C1	M1	A1	N1	T1	E1	H1
RVA/Human-tc/IND/mani-97/2006/G9P[19]	**G9**	**P[19]**	**I5**	R1	C1	M1	**A8**	N1	T1	E1	H1
RVA/Human-wt/BGD/Dhaka6/2001/G11P[25]	**G11**	**P[25]**	I1	R1	C1	M1	A1	N1	T1	E1	H1
RVA/Human-wt/VNM/30378/2009/G26P[19]	**G26**	**P[19]**	**I5**	R1	C1	M1	**A8**	N1	T1	E1	H1
RVA/Human-wt/BRA/rj24598/2015/G26P[19]	**G26**	**P[19]**	**I5**	R1	C1	M1	**A8**	N1	T1	E1	H1

Blue shading indicates the gene segments with genotypes identical to those of UFS-NGS-MRC-DPRU4723. Bold font indicates genotypes associated with porcine strains. “−” indicates that no sequence data were available in GenBank/EMBL/DDBJ data banks. * Genotype assignment based on reports by [37] (strain 03-98sP50) and (strain BG260) [46]. To date, the nucleotide accession numbers for the 11 gene segments of strains 03-98sP50 and BG260 are not available in the GenBank, EMBL, or DDBJ data banks.

## Supplementary Materials

The following are available online at https://www.mdpi.com/2076-0817/9/8/663/s1. Supplementary data 1 (S1): Identity matrices for the VP1, VP2, VP3, VP4, VP6, VP7, NSP1, NSP2, NSP3, NSP4, and NSP5 nucleotide and deduced amino acid identities among strains calculated using the p-distance algorithm in MEGA 6.06. Supplementary data 2: Comparison of amino acid sequences. Supplementary data 3: Additional phylograms of the VP1, VP2, VP3, NSP1, NSP2, NSP3, NSP4 and NSP5.
