# Supplementary material for: Molecular Characterisation of a Rare Reassortant Porcine-Like G5P[6] Rotavirus Strain Detected in an Unvaccinated Child in Kasama, Zambia
Source: Pathogens. 2020 Aug 17;9(8):663. doi: 10.3390/pathogens9080663 (PMC7460411; doi:10.3390/pathogens9080663)
Supplement: Supplementary file 1 [file pathogens-09-00663-s001.zip › supplementary data/Supplementary data 2-comparison of aminoacid sequences.docx]

**Supplementary data 2a.** Comparison of the deduced amino acid sequence of gene segment 9 of strain RVA/Human-wt/ZMB/UFS-NGS-MRC-DPRU4723/2014/G5P[6] to a selection of human and animal G5 sequences obtained from the GenBank. Only amino acids which differ are shown. Variable regions designated VR-1 to VR-9 are shown. The dots (•) represents conserved amino acids relative to the study strain. The dashes (-) indicate the absence of amino acid residue in that location.

**Supplementary data 2b**. Comparison of the deduced amino acid sequence of gene segment 4 of strain RVA/Human-wt/ZMB/UFS-NGS-MRC-DPRU4723/2014/G5P[6] to a selection of older and contemporary P[6] sequences obtained from the GenBank. Only amino acids which differ are shown. Variable regions designated VR-1 to VR-9 are shown. * are regions in which amino acid substitution has been found in mutants selected with NMAbs. The dots (•) represent conserved amino acids relative to the study strain. The dashes (-) indicate the absence of amino acid residue in that location.
