# Supplementary material for: Molecular Characterisation of a Rare Reassortant Porcine-Like G5P[6] Rotavirus Strain Detected in an Unvaccinated Child in Kasama, Zambia
Source: Pathogens. 2020 Aug 17;9(8):663. doi: 10.3390/pathogens9080663 (PMC7460411; doi:10.3390/pathogens9080663)
Supplement: Supplementary file 1 [file pathogens-09-00663-s001.zip › supplementary data/Supplementary data 3 - phylograms.docx]

**Supplementary data 3a**. Phylogenetic tree constructed from the nucleotide sequences of the VP1 genes of strain RVA/Human-wt/ZMB/UFS-NGS-MRC-DPRU4723/2014/G5P[6] and representative strains. The position of strain RVA/Human-wt/ZMB/UFS-NGS-MRC-DPRU4723/2014/G5P[6] is shown by the black square (▪). Reference strains obtained from GenBank are represented by Accession number, Strain name, Country and year of isolation. The three closest strains as identified by BLASTn are also included. Bootstrap values ≥70% are shown adjacent to each branch node. Scale bar: 0.05 substitutions per nucleotide.

**Supplementary data 3b**. Phylogenetic tree constructed from the nucleotide sequences of the VP2 genes of strain RVA/Human-wt/ZMB/UFS-NGS-MRC-DPRU4723/2014/G5P[6] and representative strains. The position of strain RVA/Human-wt/ZMB/UFS-NGS-MRC-DPRU4723/2014/G5P[6] is shown by the black square (▪). Reference strains obtained from GenBank are represented by Accession number, Strain name, Country and year of isolation. The three closest strains as identified by BLASTn are also included. Bootstrap values ≥70% are shown adjacent to each branch node. Scale bar: 0.05 substitutions per nucleotide.

**Supplementary data 3c**. Phylogenetic tree constructed from the nucleotide sequences of the VP3 genes of strain RVA/Human-wt/ZMB/UFS-NGS-MRC-DPRU4723/2014/G5P[6] and representative strains. The position of strain RVA/Human-wt/ZMB/UFS-NGS-MRC-DPRU4723/2014/G5P[6] is shown by the black square (▪). Reference strains obtained from GenBank are represented by Accession number, Strain name, Country and year of isolation. The three closest strains as identified by BLASTn are also included. Bootstrap values ≥70% are shown adjacent to each branch node. Scale bar: 0.05 substitutions per nucleotide.

**Supplementary data 3d**. Phylogenetic tree constructed from the nucleotide sequences of the NSP1 genes of strain RVA/Human-wt/ZMB/UFS-NGS-MRC-DPRU4723/2014/G5P[6] and representative strains. The position of strain RVA/Human-wt/ZMB/UFS-NGS-MRC-DPRU4723/2014/G5P[6] is shown by the black square (▪). Reference strains obtained from GenBank are represented by Accession number, Strain name, Country and year of isolation. The three closest strains as identified by BLASTn are also included. Bootstrap values ≥70% are shown adjacent to each branch node. Scale bar: 0.05 substitutions per nucleotide.

**Supplementary data 3e**. Phylogenetic tree constructed from the nucleotide sequences of the NSP2 genes of strain RVA/Human-wt/ZMB/UFS-NGS-MRC-DPRU4723/2014/G5P[6] and representative strains. The position of strain RVA/Human-wt/ZMB/UFS-NGS-MRC-DPRU4723/2014/G5P[6] is shown by the black square (▪). Reference strains obtained from GenBank are represented by Accession number, Strain name, Country and year of isolation. The three closest strains as identified by BLASTn are also included. Bootstrap values ≥70% are shown adjacent to each branch node. Scale bar: 0.05 substitutions per nucleotide.

**Supplementary data 3f**. Phylogenetic tree constructed from the nucleotide sequences of the NSP3 genes of strain RVA/Human-wt/ZMB/UFS-NGS-MRC-DPRU4723/2014/G5P[6] and representative strains. The position of strain RVA/Human-wt/ZMB/UFS-NGS-MRC-DPRU4723/2014/G5P[6] is shown by the black square (▪). Reference strains obtained from GenBank are represented by Accession number, Strain name, Country and year of isolation. The three closest strains as identified by BLASTn are also included. Bootstrap values ≥70% are shown adjacent to each branch node. Scale bar: 0.05 substitutions per nucleotide.

**Supplementary data 3g**. Phylogenetic tree constructed from the nucleotide sequences of the NSP4 genes of strain RVA/Human-wt/ZMB/UFS-NGS-MRC-DPRU4723/2014/G5P[6] and representative strains. The position of strain RVA/Human-wt/ZMB/UFS-NGS-MRC-DPRU4723/2014/G5P[6] is shown by the black square (▪). Reference strains obtained from GenBank are represented by Accession number, Strain name, Country and year of isolation. The three closest strains as identified by BLASTn are also included. Bootstrap values ≥70% are shown adjacent to each branch node. Scale bar: 0.05 substitutions per nucleotide.

**Supplementary data 3h**. Phylogenetic tree constructed from the nucleotide sequences of the NSP5 genes of strain RVA/Human-wt/ZMB/UFS-NGS-MRC-DPRU4723/2014/G5P[6] and representative strains. The position of strain RVA/Human-wt/ZMB/UFS-NGS-MRC-DPRU4723/2014/G5P[6] is shown by the black square (▪). Reference strains obtained from GenBank are represented by Accession number, Strain name, Country and year of isolation. The three closest strains as identified by BLASTn are also included. Bootstrap values ≥70% are shown adjacent to each branch node. Scale bar: 0.05 substitutions per nucleotide.
